# β-Catenin Inactivation Is a Pre-Requisite for Chick Retina Regeneration

**DOI:** 10.1371/journal.pone.0101748

**Published:** 2014-07-08

**Authors:** Jie Zhu, Agustin Luz-Madrigal, Tracy Haynes, Julia Zavada, Amy K. Burke, Katia Del Rio-Tsonis

**Affiliations:** Department of Biology, Miami University, Oxford, Ohio, United States of America; University of Dayton, United States of America

## Abstract

In the present study we explored the role of β-catenin in mediating chick retina regeneration. The chick can regenerate its retina by activating stem/progenitor cells present in the ciliary margin (CM) of the eye or via transdifferentiation of the retinal pigmented epithelium (RPE). Both modes require fibroblast growth factor 2 (FGF2). We observed, by immunohistochemistry, dynamic changes of nuclear β-catenin in the CM and RPE after injury (retinectomy). β-catenin nuclear accumulation was transiently lost in cells of the CM in response to injury alone, while the loss of nuclear β-catenin was maintained as long as FGF2 was present. However, nuclear β-catenin positive cells remained in the RPE in response to injury and were BrdU-/p27+, suggesting that nuclear β-catenin prevents those cells from entering the cell cycle. If FGF2 is present, the RPE undergoes dedifferentiation and proliferation concomitant with loss of nuclear β-catenin. Moreover, retinectomy followed by disruption of active β-catenin by using a signaling inhibitor (XAV939) or over-expressing a dominant negative form of Lef-1 induces regeneration from both the CM and RPE in the absence of FGF2. Our results imply that β-catenin protects cells of the CM and RPE from entering the cell cycle in the developing eye, and specifically for the RPE during injury. Thus inactivation of β-catenin is a pre-requisite for chick retina regeneration.

## Introduction

Retina regeneration studies have been conducted in different animal models for many years, however, the molecular mechanism underlying regeneration via different cellular sources is still under rigorous investigation [1], [2], [3], [4], [5]. At Embryonic day 4 (E4, HH stage 22–24) [6] chick eyes can regenerate a complete retina upon retinectomy, as long as there is a source of growth factors present in the eye [7], [8], [9], [10], [11]. The embryonic chick can regenerate its retina via two different mechanisms: by the activation of stem/progenitor cells present in the CM and by RPE transdifferentiation. During transdifferentiation, the RPE dedifferentiates, proliferates and forms a neuroepithelium that eventually differentiates into retinal cells [8], [11]. Several signaling pathways including FGF, Sonic Hedgehog (Shh) and Bone Morphogenetic Protein (BMP), as well as inflammation molecules C3a, C5a and IL-6 have been shown to be involved in chick retina regeneration [7], [8], [9], [10], [11], [12], [13]; however the molecular mechanism underlying both regeneration processes still needs to be further explored.

β-catenin is a dual function protein, playing a critical role in cell-cell adhesion as well as mediating gene transcription through Wnt signaling [14], [15]. Overexpression of active β-catenin during early chick eye development (E1.5; HH stage 10) promotes retinal cells to change their fates and gain peripheral identity [16]. β-catenin is also required for RPE specification during avian and mammalian eye development by directly regulating two RPE-specific genes, Microphthalmia-associated transcription factor (Mitf) and orthodenticle homolog 2 (Otx2). Disruption of β-catenin causes the RPE to lose its phenotype and to start to express retinal progenitor markers, while the overexpression of β-catenin and Otx2 is sufficient to induce Mitf expression in retinal progenitors [17], [18], [19].

The transcriptional functions of β-catenin can be regulated by Wnt signaling which controls β-catenin levels in the cytoplasm by modulating phosphorylation events. In the absence of Wnt signaling, the β-catenin destruction complex continuously phosphorylates β-catenin protein at Ser 33, 37, 45 and Thr 41 to target β-catenin for degradation via an ubiquitin-dependent pathway [20], [21], [22]. Upon stimulation, Wnt ligands bind to membrane receptor Frizzled and co-receptor LRP5/6, triggering a downstream signaling cascade leading to the inactivation of the destruction complex and the stabilization of β-catenin which is no longer phosphorylated at the same residues. The stabilized β-catenin is then targeted for nuclear translocation [23]. It has been shown that in chick neural cells, phosphorylation of β-catenin at tyrosine 489 (Y489) targets it to the nucleus where it binds to its partner(s) TCF/Lef1 and acts as a co-activator of transcription [24]. In this complex, β-catenin functions as a transcriptional co-activator to facilitate the binding of the complex to chromatin and also to recruit components to promote chromatin remodeling [25], [26].

Since β-catenin has been reported to be important for the development or maintenance of the RPE and CM, we sought to explore the role of β-catenin in these tissues during retina regeneration. Here, we report that active β-catenin is associated with cells having a low proliferative status in the CM and RPE and that blocking the transcriptional activity of β-catenin is a necessary step in retina regeneration.

## Materials and Methods

### Chick embryos

Fertilized Specific Pathogen Free (SPF) chicken eggs (Charles River Laboratories, Wilmington, MA, USA) were incubated in a humidified rotating incubator at 38°C.

### Surgical procedures

Retinectomies were performed in eyes of chick embryos at E4 (HH stage 22–24) using fine forceps as previously described [8], [9]. For FGF2 treated eyes, heparin beads (Sigma, St. Louis, MO, USA) containing FGF2 (R&D System, Minneapolis, MN, USA) were prepared and added to the eyes as described in [9]. For the inhibitory experiments, 5 ul of 2 mM Wnt/β-catenin signaling inhibitor XAV939 (Reagents Direct, Encinitas, CA, USA) or PBS was injected into the vitreous chamber 30 minutes post-retinectomy. Embryos were collected 1, 3 or 7 days post-retinectomy (d PR) and processed as described below for histology and immunohistochemistry.

### Retroviral construct generation

RCAS DN-Lef1-HA was generated using the Gateway system as previously described [27]. Chicken *lef1* (NM_205013) cDNA was synthetized by RT-PCR using RNA from the CM at E4 followed by PCR amplification of the partial sequence of *lef1* using the primers listed in Table S1 in File S1. The amplified fragment was cloned in pENTR-TOPO vector (Invitrogen, Carlsbad, CA, USA) to generate pENTR DN-Lef1 followed by recombination with RCAS BPA-CHA (Addgene, Cambridge, MA, USA) using Gateway LR-Clonase II Enzyme Mix (Invitrogen, Carlsbad, CA, USA). Retroviral stocks were generated and amplified using DF-1 cells (ATCC CRL-12203) and the titer was determined using the antibody AMV-3C2 (1∶100, Hybridoma bank, Iowa City, Iowa, USA) against the viral gag protein.

### *In vitro* electroporation

CM explants were isolated from E4 embryos as previously described [13] and placed in 1 ml of 1X Hank's Balanced Salt Solution (HBSS). For the electroporation, the HBSS was removed and 8–10 CM explants per biological sample were mixed with 1 µl containing 2 µg of RCAS DN-Lef1-HA or RCAS-GFP constructs and placed between two platinum-iridium electrodes using the same system previously described [28]. Electroporated CM explants were cultured for 48 hours in 24-well plates in culture medium [10], [16] containing 10% FCS, 45% HAMS F12 nutrient, 45% DMEM, 2.5 mM L-Glutamine, Penicillin 100 unit/ml, streptomycin 100 µg/ml, 15 mM HEPES at 37 °C and 5% CO_2_. The RCAS DN-Lef1-HA infection was confirmed by RT-PCR using specific primers listed in Table S1 in File S1.

### Delivery of RCAS and electroporation

RCAS DN-Lef1-HA or RCAS-GFP (a kind gift from Teri Belecky-Adams, IUPUI, Indianapolis, IN, USA) were injected into the vitreous cavity or sub-retinal space for CM or RPE infection respectively in E3 (HH Stage 19–20) embryos. The embryos were collected at 3 and 7 d PR and the viral infection was confirmed by immunohistochemistry using the AMV-3C2 antibody. Electroporation was performed also in E3 embryos as described in [29] with some modifications. A small window was made in the shell, 100–200 µl of 1X HBSS were applied over the embryo followed by injection of 1 µl (2 µg) of RCAS DN-Lef1-HA or RCAS-GFP constructs in the optic cup using a Pico-injector system PLI-100 (Harvard Apparatus, Holliston, MA, USA) and glass capillary needles. A gold plated wire electrode used as anode (BTX, Bent, Holliston, MA, USA, in ovo gene Model 512) was placed in the ventral border of the eye. A platinum/iridium electrode (FHC Inc, Bowdoinham, ME, USA) was used as cathode and was inserted on the top of the brain. Electroporation conditions were the same as those described in the *in vitro* electroporation [28]. The window was sealed and the embryos were incubated until desired collection time.

### Cell proliferation

Thirty microliters of 10 mg/ml BrdU (Roche, Indianapolis, IN, USA) solution was injected over the eyes of embryos 1 hour before collection. Tissues were fixed and processed for immunohistochemistry.

### Histology

Tissues used for histology were fixed in Bouin's solution fixative (Ricca Chemical Company, Arlington, TX, USA) for 1 hour and transferred into 70% ethanol until embedded in paraffin wax. The tissues were sectioned and stained with hematoxylin and eosin. Histological results are representative of 10 or more eyes. Histological images were photographed using an Olympus BX-51 microscope (Tokio, Japan).

### Immunohistochemistry

Tissues used for immunohistochemistry were fixed in 4% paraformaldehyde for 1 hour, rinsed in 1x PBS and cryoprotected in 30% sucrose, then snap frozen in optimal cutting temperature medium (O.C.T.) (Tissue-Tek, Sakura Finetek, Torrance, CA, USA). The frozen tissues were sectioned at 10 µm, washed in 1x PBS and blocked in serum for 1 hour. When antibodies against transcription factors were used, a 10-minute 1% saponin (Sigma, St. Louis, MO, USA) permeabilization was used followed by three washes in 1x PBS. Primary antibodies (Table S2 in File S1) diluted in blocking solution were added to sections and incubated overnight at 4°C, followed by washes in 1x PBS and incubation with secondary antibody for at least 2 hours at room temperature. Excess secondary antibody was washed off with 1x PBS and the slides were mounted in Vectashield mounting medium (Vector Labs, Burlingame, CA, USA) with coverslips. Secondary antibodies against the host species of listed primary antibodies were used (Molecular Probes, Invitrogen, Carlsbad, CA, USA). For negative controls either the primary antibody was omitted and only the secondary antibody was used or an isotype specific IgM (for β-catenin) was used instead of the primary antibody. Confocal images (size 1024×1024) were collected sequentially on Olympus FV500 Laser Scanning Confocal System (Tokio, Japan).

### Reverse transcriptase quantitative PCR (RT-qPCR)

Total RNA was extracted from the CM explants electroporated with RCAS constructs (see in vitro electroporation) using Nucleospin RNA II isolation kit (Macherey-Nagel, Düren, Germany) following the manufacture's protocol. The quality and quantity of RNA was determined using Agilent RNA 6000 nano LabChip (Agilent 2100 Bioanalyzer, Agilent Technologies, Santa Clara, CA, USA). In general, samples with RIN >9 were used for RT-qPCR experiments. Approximately 200 ng of total RNA were used for cDNA synthesis using ImProm-II Reverse Transcription System (Promega, Madison, WI, USA) and random-primer hexamers according to the manufacturer's instructions. RT-qPCR reactions and conditions were performed as previously described [13]. Splice junction specific primers were optimized following guidelines for RT-qPCR experiments including the PCR amplification efficiency and melting curves [30]. All primers were designed using Primer 3 (v 4.0) (http://primer3.wi.mit.edu/) and the sequences and Ensembl or GenBank IDs are provided as well as the primer efficiency for each set in Table S3 in File S1. The comparative cycle at threshold (2^−ΔΔCt^) was used to determine relative changes in transcript levels compared to *gapdh* mRNA levels as previously reported [31]. An unpaired Student's t-test analysis was calculated using SigmaPlot 8.0 Software. All analyses were performed in triplicate with at least three independent biological samples.

### Quantification

ImagePro Plus software (Media Cybernetics, Bethesda, MD, USA) was used for all quantitative analyses of the histology data. The area of regenerated neuroepithelium (in histological sections) was outlined and the average area for each treated eye was graphed. Exact two-sample permutation tests were used to determine statistical significance. Error bars represent SEM. The alpha level was set at 0.05.

Quantitation of BrdU-positive cells per 10,000 µm^2^ in regenerated neuroepithelium/retina was determined by counting 3 different areas of the regenerating neuroepthelium/retina derived from the CM and from RPE transdifferentiation in each treated eye. Three different eyes were included. Split-plot ANOVA was used to determine statistical significance between experimental and control groups. Error bars represent SEM.

## Results

### Nuclear β-catenin is detected in a subpopulation of low to non-proliferative cells of the CM and RPE during development

**Figure 1:**
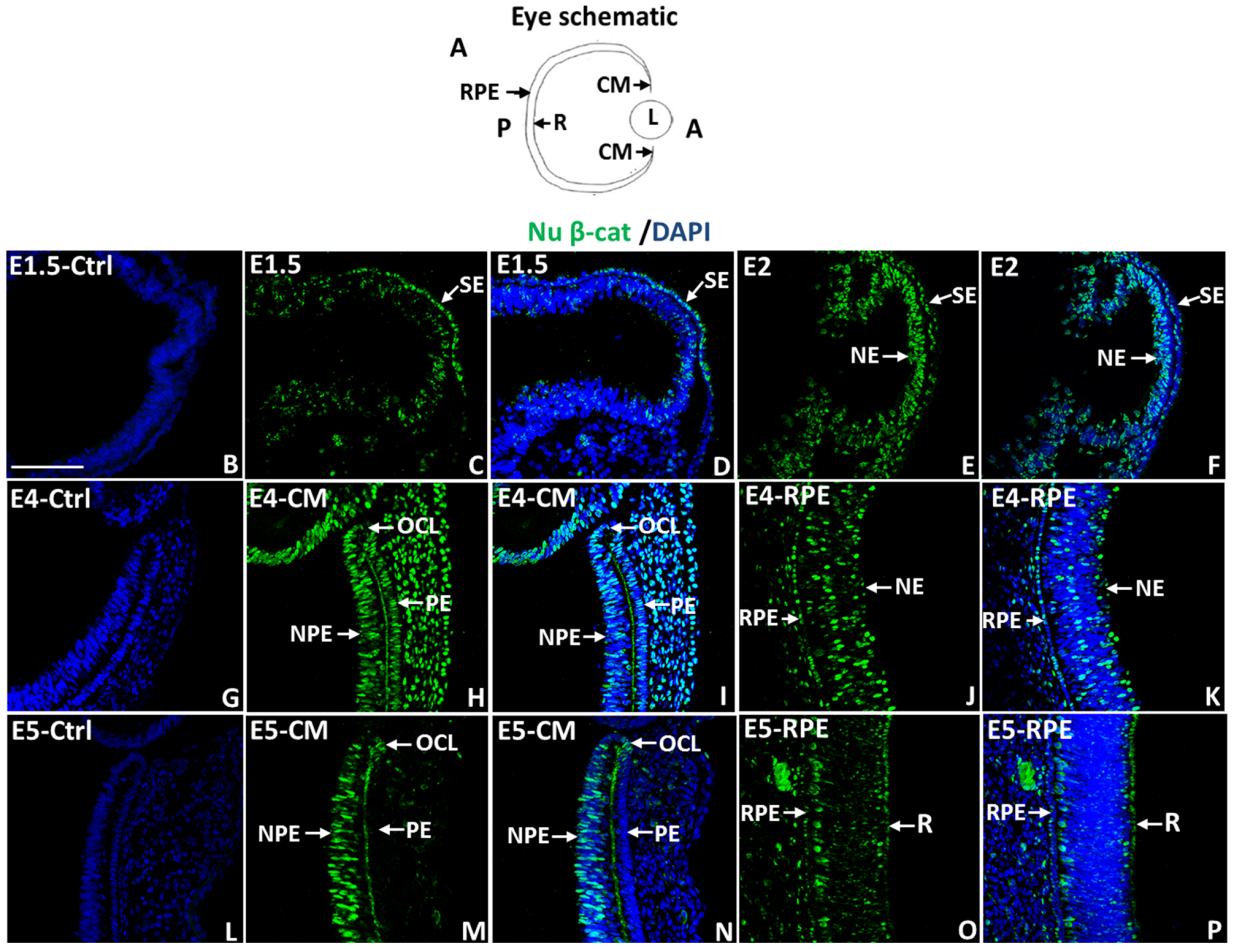
Nuclear β-catenin presence during chick eye development. (A) Schematic diagram of the eye showing the anterior (A) and posterior (P) region of the eye as well as the location of the ciliary margin (CM), retina (R), retinal pigmented epithelium (RPE) and Lens (L). The orientation of the eye in the diagram applies to all images in this paper. (B–F) Immunohistochemistry showing the location of nuclear β-catenin (Nu β-cat) in the developing optic vesicles at E1.5 (HH 10) (B–D) and E2 (HH 13) (E–F). (G–P) Nu β-cat presence in the developing CM and RPE at E4 (HH 22–24) (G–K) and E5 (HH 27–28) (L–P). DAPI stains the nuclei of the cells in (B, D, F, G, I, K, L, N, P). B, G and L are the corresponding negative controls for E 1.5 (B), E4 (G) and E5 (L). SE: surface Ectoderm; NE: neuroepithelium; NPE: non-pigmented epithelium; PE: pigmented epithelium. OCL: optic cup lip. The scale bar in (B) represents 100 µm and applies to all panels.

**Figure 2:**
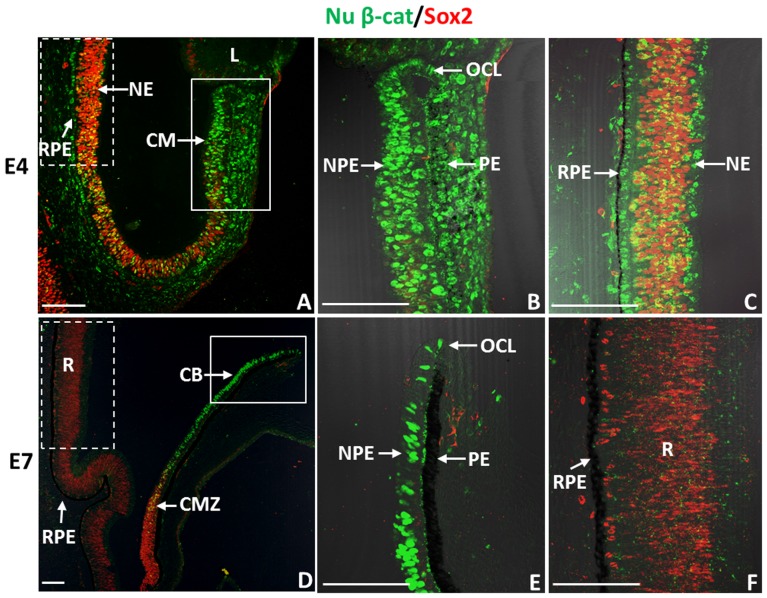
Nuclear β-catenin^+^ cells in the CM and RPE of chick eyes do not co-express Sox2. (A) Immunohistochemistry shows patterns of nuclear β-catenin (Nu β-cat)/Sox2 in E4 eyes. (B) Close-up image of the CM (boxed solid line) area in (A). (C) Close-up image of the RPE (boxed dash line) area in (A). (D) Patterns of Nu β-cat/Sox2 in E7 eyes. (E) Close-up image of the boxed (solid line) area in (D). (F) Close-up image of the boxed (dash line) area in (D). CM: ciliary margin; NPE: non-pigmented epithelium; PE: pigmented epithelium; OCL: optic cup lip; CB: ciliary body; CMZ: ciliary marginal zone; RPE: retinal pigmented epithelium; NE: neuroepithelium; R: retina. The scale bars represent 100 µm in all panels.

Presence of Wnt signaling molecules and activation of Wnt signaling in the CM and RPE during chick eye development has been shown by *in situ* hybridization and reporter assays [16], [32], [33], [34], [35], [36]. We decided to use an antibody that specifically recognizes the transcriptionally active form of β-catenin (PY-489-β-catenin) [24] to investigate its temporal and spatial distribution during chick eye development and regeneration. During initial formation of the optic vesicle, nuclear β-catenin is mostly present in the surface ectoderm (E1.5; HH Stage10; Fig. 1C, D) with minor presence in the neuroepithelium (NE). By late optic vesicle formation (E2, HH Stage 13), nuclear β-catenin is still present in the surface ectoderm (SE) but is more prominent in the NE (Fig. 1E, F). At E4 (HH Stage 24; stage at which retinectomies are performed for regeneration studies), nuclear β-catenin immunoreactivity was detected in a subpopulation of cells in the CM including the non-pigmented epithelium (NPE) connected to the retina posteriorly, and the pigmented epithelium (PE) connected to the RPE posteriorly (Fig. 1H, I). The nuclear β-catenin^+^ NPE cells extended to the optic cup lip (OCL), the most anterior domain of the CM that has been reported to house multipotent optic cup stem/progenitor cells including ones for the retina [37], [38]. The nuclear β-catenin^+^ area is defined by collagen IX staining, a widely used marker for the anterior region of the CM known as the ciliary body, that has been shown to contain retinal stem cells [33], [39], [40], [41] (File S1). Some, but not all the cells of the RPE at E4 were also nuclear β-catenin^+^ (Fig. 1J, K). At E5 (HH stage 27–28) and E7 (HH stage 31–32), nuclear β-catenin was only detected in a population of cells located in the NPE including the OCL, while the PE and the RPE showed low or no nuclear β-catenin (Fig. 1 M-P and Fig. 2 D–F). Therefore, as development continues nuclear β-catenin is limited to a defined cell niche of the CM.

We then examined if nuclear β-catenin^+^ cells found in the CM and the RPE of developing eyes co-express Sox2, a widely used marker for proliferating retinal progenitor cells during eye development [42], [43]. Interestingly, nuclear β-catenin^+^ cells of the NPE and PE of the CM, as well as cells of the RPE rarely co-expressed Sox2 (Sox2^-^) at E4 or E7 (Fig. 2 A, B, D, E). Some Sox2^+^/nuclear β-catenin^+^ cells were seen in the developing NE at E4 but by E7, Sox2 was predominantly expressed in the differentiating retina and the adjacent ciliary marginal zone (CMZ), the posterior region of the CM, where retinal progenitors are located [38], [43] (Fig. 2 A, C, D and F).

**Figure 3:**
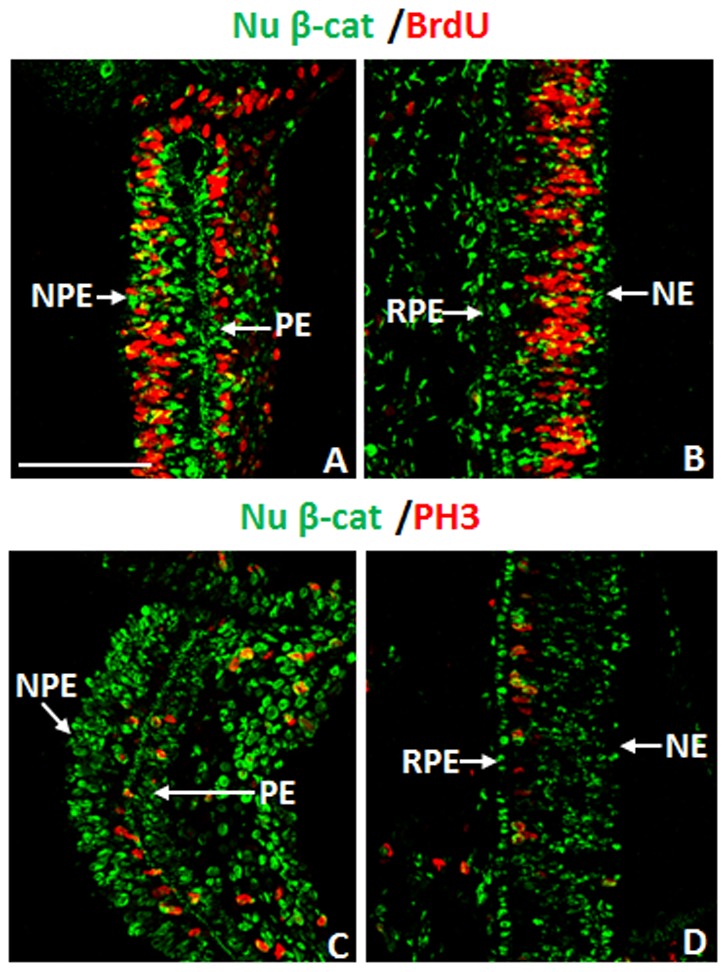
Many nuclear β-catenin^+^ cells in the CM and RPE of E4 chick eyes are not proliferating. (A–B) The presence of nuclear β-catenin (Nu β-cat) and BrdU incorporation in the CM (A) and RPE (B) at E4. (C–D) Double immunohistochemistry for Nu β-cat and PH3 in the CM (C) and RPE (D) at E4. NPE: non-pigmented epithelium; PE: pigmented epithelium; RPE: retinal pigmented epithelium; NE: neuroepithelium. The scale bar in (A) represents 100 µm and applies to all panels.

To analyze if the nuclear β-catenin^+^ cells were actively cycling and entering the S phase, we added BrdU to the eyes one hour before collection, and subsequently performed immunohistochemistry for BrdU and nuclear β-catenin. Many of the nuclear β-catenin^+^ cells in the NPE, PE and in the RPE of E4 eyes were BrdU negative (BrdU^-^) (Fig. 3 A, B). Although a small portion of cells in the CM were phosphohistone -3 (PH3)+, few cells co-stained with nuclear β-catenin. In addition there were no nuclear β-catenin^+^ cells in the E4 RPE that co-labelled with PH3 (Fig. 3 C–D), a label for proliferating cells going through late G2-M phase of the cell cycle [44]. Overall, nuclear β-catenin is present in low or non-proliferative cells of the CM and RPE at this stage of eye development.

### The nuclear β-catenin pattern is disrupted in the CM and RPE of chick eyes after injury

**Figure 4:**
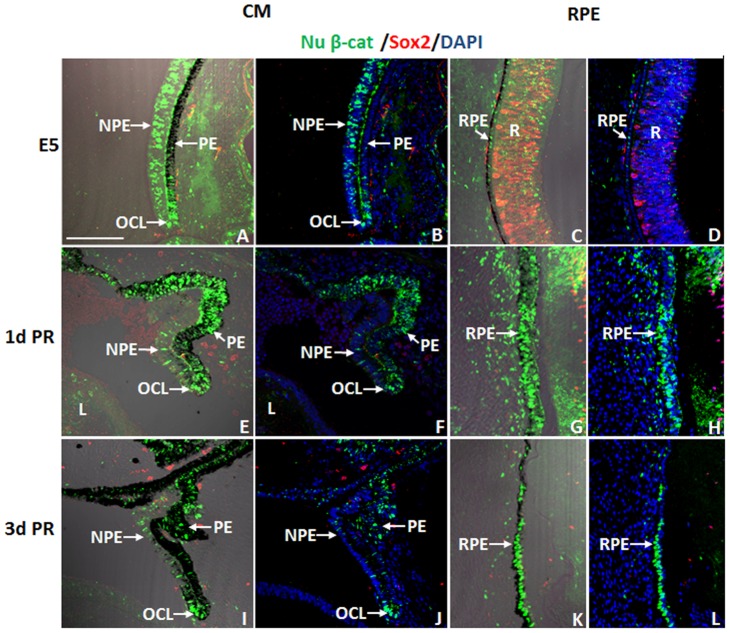
Dynamic changes of nuclear β-catenin in the chick eye after injury. (A–L) Presence of nuclear β-catenin (Nu β-cat) and Sox2 in the CM (A, B, E, F, I, J) and RPE (C, D, G, H, K, L) at E5 (A–D), at 1 d PR (E–H) and at 3 d PR (I–L). Panels A, C, E, G, I, K include DIC overlay and are equivalent to B, D, F, H, J, and L respectively. NPE: non-pigmented epithelium; PE: pigmented epithelium; OCL: optic cup lip; RPE: retinal pigmented epithelium; R: retina. DAPI stains the nuclei of the cells in B, D, F, H, J and L. Scale bar in (A) represents 100 µm and applies to all panels.

**Figure 5:**
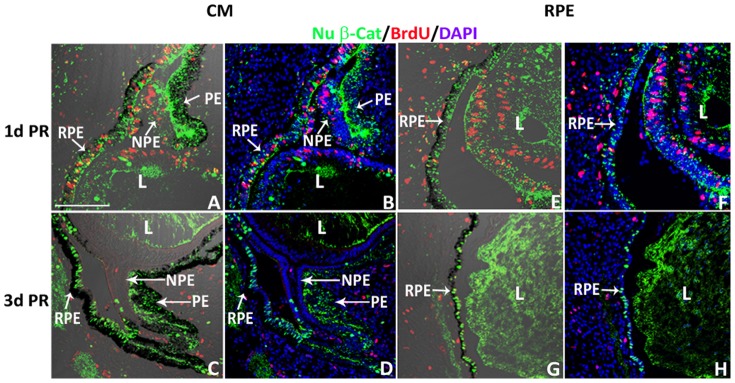
CM and RPE cells show low to no proliferative activity after retinectomy. (A–H) Presence of nuclear β-catenin (Nu β-cat) and BrdU incorporation in the CM (A–D) and RPE (E–H) at 1 d PR (A, B, E, F) and 3 d PR (C, D, G, H). Panels A, C, E, G contain DIC overlay and are equivalent to B, D, F, and H respectively. NPE: non-pigmented epithelium; PE: pigmented epithelium; RPE: retinal pigmented epithelium; L: lens. DAPI stains the nuclei of cells in B, D, F and H. Scale bar in (A) represents 100 µm and applies to all panels.

**Figure 6:**
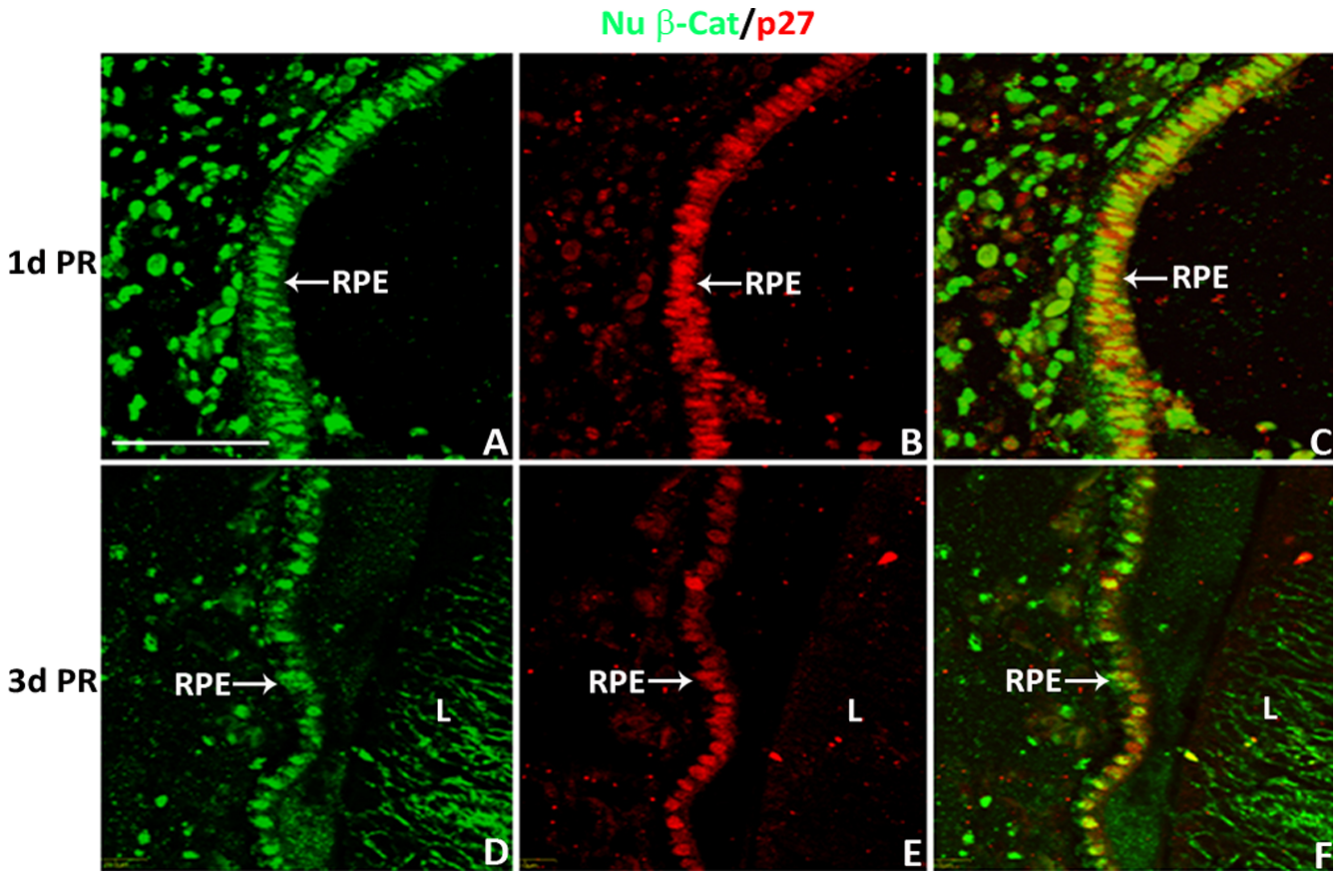
Nuclear β-catenin^+^ RPE cells co-localize with cell cycle inhibitor p27 at 1 and 3 d PR. (A–F) Presence of nuclear β-catenin (Nu β-cat) and p27 at 1 d PR (A–C) and 3 d PR (D–F). RPE: retinal pigmented epithelium; L: lens. Scale bar in (A) represents 50 µm and applies to all panels.

To investigate if the nuclear β-catenin expression pattern changes with injury or during regeneration, we examined the pattern of nuclear β-catenin in the CM and the RPE at 1 and 3 d PR in the absence of any growth factors (a non-regenerative injury model). A clear reduction of nuclear β-catenin was detected in the NPE at 1 and 3 d PR (Fig. 4 E–F and I–J) compared to that of the intact eyes at equivalent developmental stages, E5 and E7 (Fig. 4 A–B and Fig. 2 D–E). Interestingly, cells in the OCL as well as cells of the PE and RPE showed increased presence of nuclear β-catenin at both 1 and 3 d PR (Fig 4 E–L) compared with the intact eyes (Fig. 4 A–D and Fig. 2 E–F). The nuclear β-catenin^+^ cells of the PE and RPE were Sox2^-^ at 1 and 3 d PR (Fig. 4 E–L). While few cells of the RPE (located towards the anterior region of the eye) did show BrdU incorporation at 1 d PR (Fig. 5 A–B) compared to 3 d PR (Fig. 5 C–H), most of the nuclear β-catenin^+^ cells of the RPE did not incorporate BrdU at 1 d PR (Fig. 5 E–F). Instead, p27, a cell cycle inhibitor that prevents cells from entering the S phase [45], co-localized with the majority of nuclear β-catenin^+^ cells of the RPE at 1 and 3 d PR (Fig. 6 A–F).

### Nuclear β-catenin is significantly down-regulated in FGF-induced retinas

**Figure 7:**
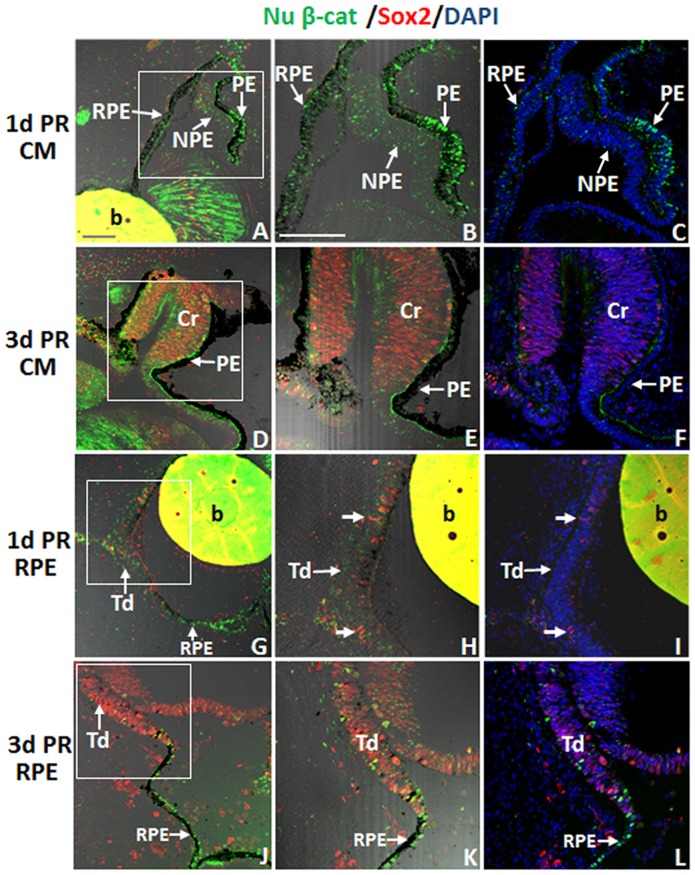
Nuclear β-catenin is absent in the CM and RPE during FGF2-induced regeneration. Co-expression of nuclear β-catenin (Nu β-cat) and Sox2 in the CM (A–F) and RPE (G–L) at 1 d PR (A–C and G–I) and 3 d PR (D–F and J–L); (B,C) show the boxed area in (A); (E, F) show the boxed area in (D); (H, I) show the boxed area in (G); and (K, L) show the boxed area in (J). Panels A, D, G, and J have DIC overlay. Panels B, E, H, and K include DIC overlay and are equivalent to C, F, I, and L respectively. b = FGF2 bead; Cr = ciliary regeneration; Td = transdifferentiation; PE: pigmented epithelium; NPE: non-pigmented epithelium; RPE: retina pigmented epithelium; L: lens. DAPI stains the nuclei in C, F, I and L. Scale bar in (A) represents 100 µm and applies to D, G and J. Scale bar in (B) represents 100 µm and applies to C, E, F, H, I, K and L.

We further examined the patterns of nuclear β-catenin in the CM and RPE of eyes at 1 and 3 d PR in the presence of FGF2. Nuclear β-catenin was clearly down-regulated or absent in FGF2-induced regenerating neuroepithelium from the CM and from the RPE (Fig. 7). At 1 d PR, in the presence of FGF2, the NPE cells showed a clear reduction of nuclear β-catenin, and an elevation of nuclear β-catenin in the PE (Fig. 7 A–C). The RPE that was close to the FGF2 heparin bead (source of growth factor) started losing pigmentation and showed a clear reduction of nuclear β-catenin staining and induction of Sox2 (Fig. 7 G–I). However, the pigmented RPE was nuclear β-catenin^+^ and Sox2^-^ (Fig. 7 G). At 3 d PR, the neuroepithelium that regenerated from the CM and RPE showed strong Sox2 immunoreactivity and loss of nuclear β-catenin (Fig. 7 D–F and J–L). The non-transdifferentiated RPE remained nuclear β-catenin^+^ (Fig. 7 K–L). Therefore, FGF2, as it induces retina regeneration, it also affects the localization and presence of β-catenin.

### Inhibition of β-catenin/TCF/Lef1 transcriptional activity is sufficient to induce chick retina regeneration

**Figure 8:**
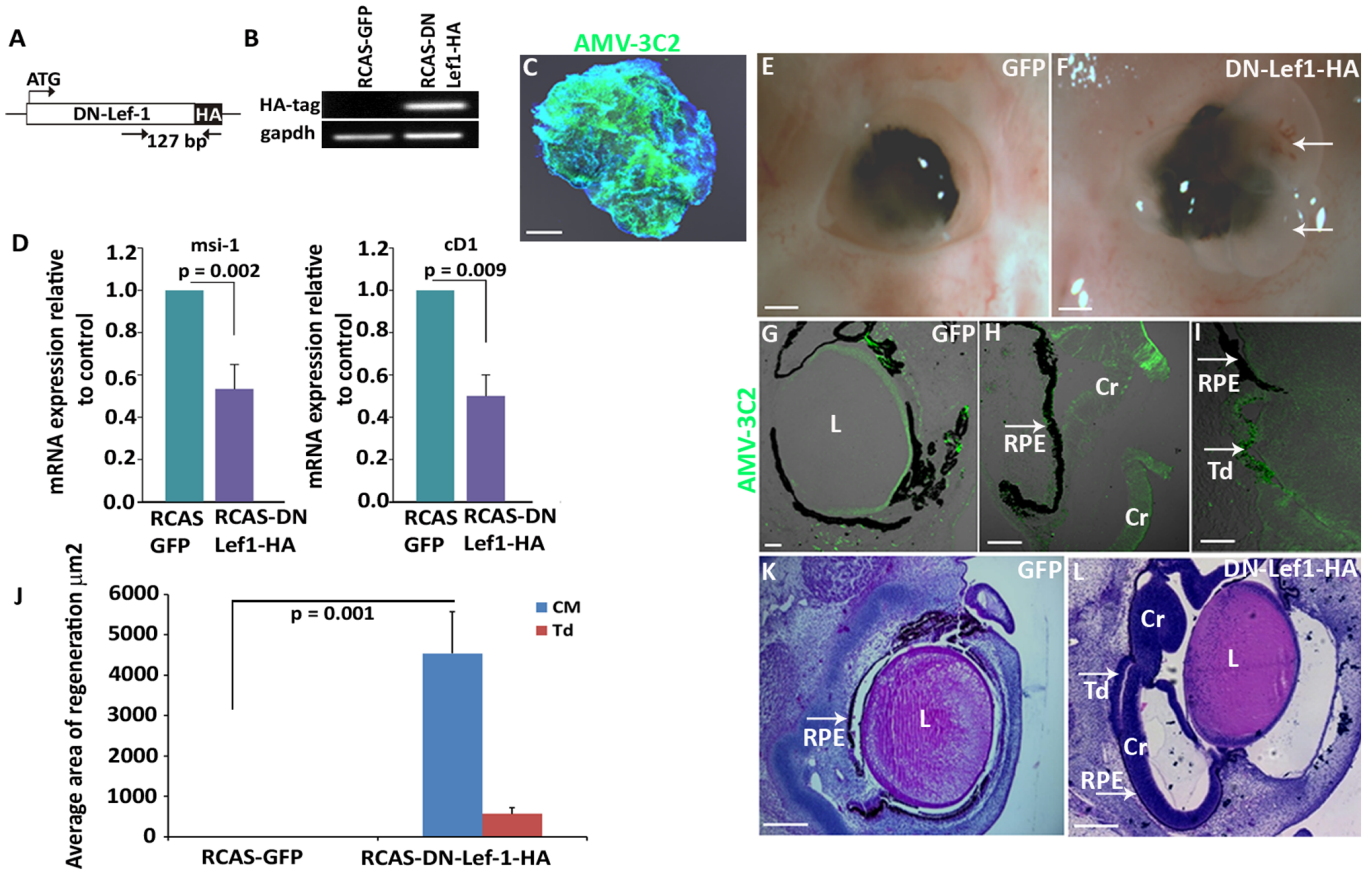
Inhibiting β-catenin/LEF/TCF transcriptional activity is sufficient to induce chick retina regeneration. (A) Schematic of a dominant negative *lef1* gene that was cloned into an RCAS vector. (B) RT-PCR confirms the RCAS DN-Lef1-HA construct is successfully expressing the HA tag in electroporated CM explants from E4 eyes. The amplified 127 bp region is shown in (A). (C) AMV-3C2 immunohistochemistry shows the presence of viral protein in RCAS DN-Lef1-HA electroporated CM explants after 48 hours. (D) RT-qPCR data shows the level of β-catenin/Lef1/TCF target genes Musashi-1 (*msi1*) and cyclin D1 (*cD1*) in RCAS DN-Lef1-HA electroporated CM explants compared to the RCAS GFP electroporated controls (*p* values shown represent significance). (E–F) Whole eye images show the amount of regeneration in the presence of RCAS GFP (E) and RCAS DN-Lef1-HA (F) at 3 d PR; arrows indicate the regenerating neuroepithelium growing out of the eye, which happens in some cases during regeneration. (G–I) AMV-3C2 immunohistochemistry shows the presence of viral proteins in RCAS GFP infected eyes (G) and in the regenerating neuroepithelium from the CM (H) and RPE transdifferentiation (I) in RCAS DN-Lef1-HA infected eyes. (J) Quantitative analysis shows the difference in amount of regeneration observed in histological sections of RCAS DN-Lef1-HA infected eyes and RCAS GFP infected eyes (*p* values shown represent significance). (K–L) Histological sections of RCAS GFP and RCAS DN-Lef1-HA infected eyes at 3 d PR. Cr = ciliary regeneration; Td = transdifferentiation; L = lens; RPE: retina pigmented epithelium. Scale bars in (C), (K) and (L) represent 200 µm; Scale bars in (E) and (F) represent 1 mm; Scale bar in (G, H and I) represents 100 µm. Error bars in (D) and (J) represent S.E.M.

The dynamic changes of β-catenin nuclear protein in developing eyes, in response to retinectomy, and during FGF-induced retina regeneration, suggest that nuclear β-catenin is involved in chick retina regeneration. It is known that nuclear β-catenin is able to bind TCF/Lef1 and activate transcription [24], [46]. Therefore, we overexpressed a dominant negative form of the chick Lef1 gene (DN-Lef1) in retinectomized chick eyes using a retroviral system to prevent the formation of β-catenin/TCF/Lef1 transcription complex and investigate if inhibiting β-catenin signaling would be sufficient to induce retina regeneration. This dominant negative form of Lef1 has the first 31 amino acids at the N-terminus deleted. It binds DNA but is unresponsive to nuclear targeted β-catenin (Fig. 8A) [47]. The retroviral DN-Lef1construct (RCAS-DN-Lef1-HA) was successfully electroporated in chick ciliary explants, and verified for expression via RT-PCR and immunohistochemistry (Fig. 8 B, C). A clear reduction of β-catenin downstream target genes Musashi-1 (*msi-1*) and cyclin D1 (*cD1*) was detected in DN-Lef1 electroporated ciliary explants verifying the activity of the construct (Fig. 8D) [48], [49], [50]. Interestingly, overexpression of the dominant negative form of Lef1 was sufficient to induce retina regeneration via the activation of CM stem/progenitor cells (14/17 eyes) and RPE transdifferentiation (5/17 eyes) (Fig. 8 F, H, I and L). Regeneration from the CM was much more robust than that of RPE transdifferentiation (Fig. 8 J). Eyes exposed to control RCAS-GFP did not show any regeneration (0/10 eyes) (Fig. 8 E, G, J and K).

### Cell proliferation in neuroepithelium/retina induced by blocking β-catenin/TCF/Lef1 transcriptional activity

**Figure 9:**
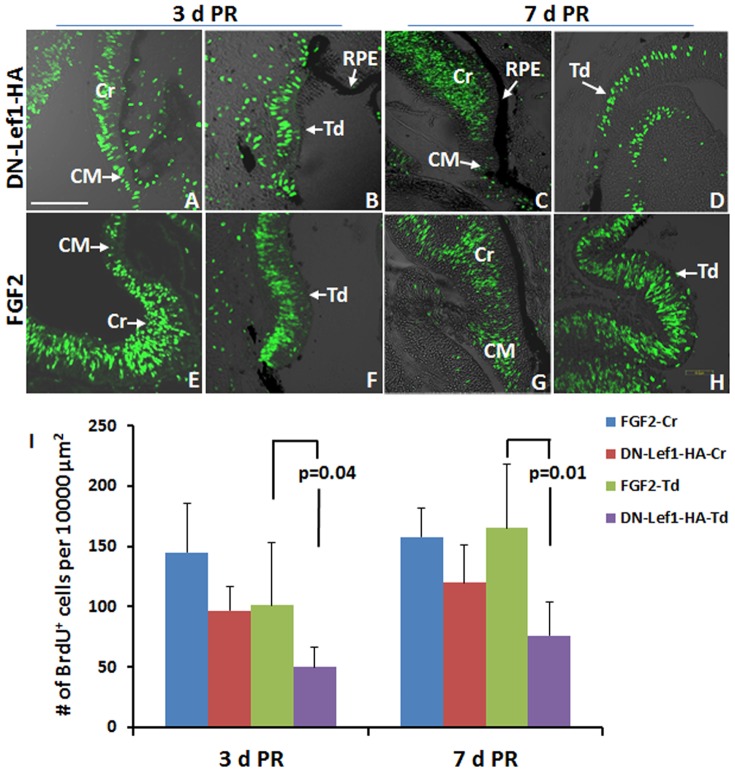
DN-Lef1 induced neuroepithelium show robust proliferation. (A–H) Immunohistochemistry shows the level of BrdU incorporation in RCAS DN-Lef1-HA (DN-Lef1-HA) infected eyes (A–D) and FGF2 treated eyes (E–H) in the new regenerate from the CM (A, C, E, G) and RPE (B, D, F, H) at 3d PR (A, B, E, F) and 7 d PR (C, D, G, H). (I) Quantification of BrdU^+^ cells in DN-Lef1-HA and FGF2-induced regenerating neuroepithelium/retina (*p* values shown represent significance). Error bars represent S.E.M. CM: ciliary margin; Cr: regeneration from ciliary margin; RPE: retinal pigment epithelium; Td: regeneration from RPE. Scale bar in (A) represents 100 µm and applies to all panels.

Robust cell proliferation was detected via BrdU incorporation in regenerating neuroepithelium/retina from the CM and from RPE transdifferentiation by blocking β-catenin/TCF/Lef1 transcriptional activity at 3 and 7 d PR (Fig. 9). Comparison of BrdU^+^ cells in DN-Lef1 and FGF2- CM-induced regenerating neuroepithelium/retina at 3 and 7 d PR showed that the proliferation activity was not significantly different, however, the DN-Lef1 induced RPE transdifferentiation showed lower proliferation activity compared to that of FGF2 at 3 d PR (p = 0.04) and 7 d PR (p = 0.01) (Fig. 9I). Most of cells in the DN-Lef1 and FGF2 induced neuroepithelium at 3 d PR were positive for Pax6 and Chx10 immunostaining, indicating those cells were retinal progenitor cells [51] (Fig. S2 A-B and E-F). At 7 d PR, the proportion of progenitors was also similar in FGF2 and DN-Lef-1 treated eyes (Fig. S2 C–D and G–H).

### Cell differentiation in DN-Lef1 induced retina

**Figure 10:**
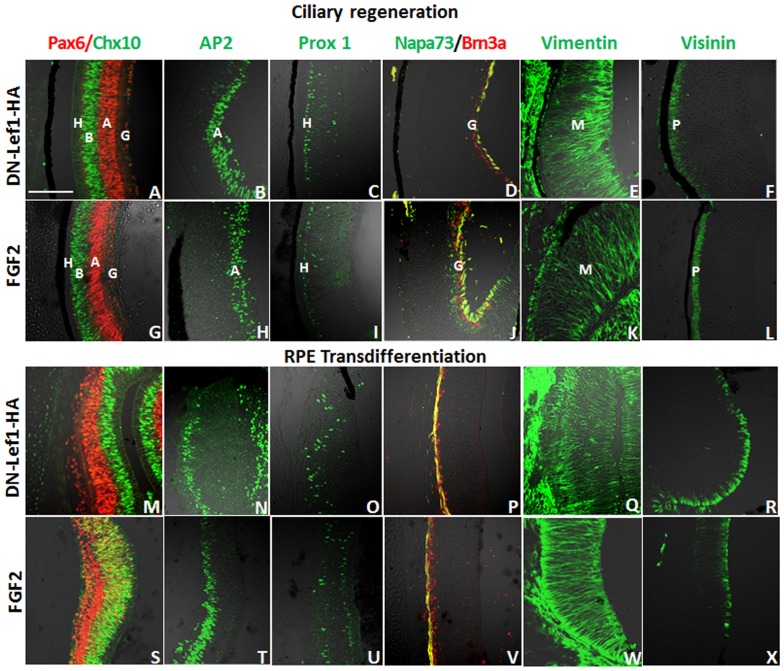
All major retinal cell types are present in DN-Lef1 induced retina at 7 d PR. (A-X) Immunohistochemistry showing retinal cell types present in DN-Lef1 induced retina from the CM (A–F) and RPE (M–R) as well as the retinal cell types present in FGF2 induced retina from the CM (G–L) and RPE (S–X) for comparison. Antibodies for Pax-6 (red) and Chx-10 (green) show the presence of ganglion (G), amacrine (A), bipolar (B), and horizontal (H) cells (A, G, M, S). Antibody for AP2 (green) shows the presence of amacrine (A) cells (B, H, N, T). Antibody for Prox-1 (green) shows the presence of horizontal (H) cells (C, I, O, U). Antibodies for Brn3a (red) and Napa-73 (green) show ganglion cells (G) and formation of ganglion axons (yellow) (D, J, P, V). Antibody for vimentin (green) shows the presence and organization of Müller (M) glia (E, K, Q, W). Antibody for visinin (green) detects photoreceptors (P) (F, L, R, X). Scale bar in (A) represents 100 µm and applies to all panels.

The retina houses neurons arranged in three cell layers. The innermost cell layer contains retinal ganglion cells, the inner nuclear layer has bipolar, amacrine, and horizontal cells, and the outer nuclear layer contains the bodies of photoreceptors. Müller glia cells expand throughout the different cell layers of the retina [52]. Antibodies recognizing proteins specifically expressed in each retinal cell type were used to determine that DN-Lef1 did indeed induce neuroepithelium from both the CM and RPE that was able to differentiate into all major retinal cell types including Müller glia cells as seen in previously studied FGF2 induction (Fig. 10) [8].

### Selective Wnt/β-catenin signaling inhibitor XAV939 induces retina regeneration

β-catenin is the key mediator of the canonical Wnt signaling pathway. Nuclear targeted β-catenin binds to the transcription factor TCF/Lef1 to induce the β-catenin/TCF/Lef1 transcription activity, which is a hallmark of Wnt/β-catenin signaling activation [24], [46]. The dynamic changes of nuclear β-catenin immunoreactivity in the stem/progenitor cells of the CM and cells of the RPE in response to retinectomy, as well as during FGF-induced retina regeneration motivated us to further investigate how this nuclear targeting of β-catenin is affected by the upstream signaling cascade of the Wnt pathway, and if the dynamic changes of nuclear β-catenin staining in the CM and RPE indeed reflect the changes of canonical Wnt signaling in those cells in response to injury and during FGF induced retina regeneration.

**Figure 11:**
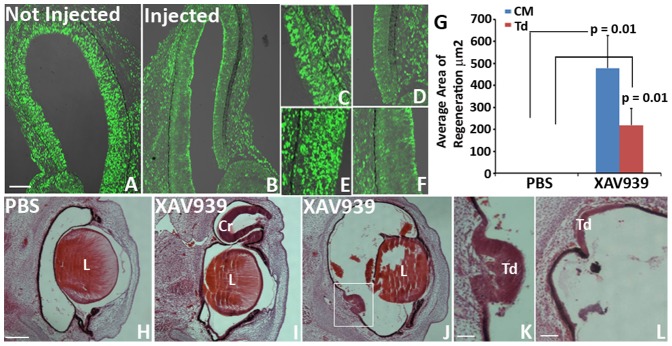
Canonical Wnt signaling inhibitor XAV939 induces chick retina regeneration. (A–F) Patterns of nuclear β-catenin (Nu β-cat) 24 hours after intravitreous injection of Wnt signaling inhibitor XAV939 into an E3 developing chick eye (B) compared to the contralateral non-injected eye (A). Close up images of the CM (C, D) and the RPE (E, F) of the non-injected eye (C, E) and the treated eye (D, F). (G) Quantitative analysis shows the difference in regeneration observed in histological sections of XAV939 treated eyes vs PBS treated eyes at 3 d PR (*p* values shown represent significance). Error bars represent S.E.M. (H–L): Histological sections of eyes treated with PBS (H) or XAV939 (I–L) 3 d PR showing regeneration from the CM (I) and transdifferentiation (J–L). (K) Shows the boxed area in (J). Cr = ciliary regeneration; Td = transdifferentiation; L = lens. Scale bar in (A) represents 100 µm and applies to (B). Scale bar in (H) represents 200 µm and applies to (I) and (J). Scale bar in (K) represents 50 µm. Scale bar in (*L*) represents 100 µm.

XAV939 is an antagonist of the Wnt/β-catenin pathway. It inhibits the enzymes tankyrase 1/2 that inhibits the activity of axin 2 (a component of the β-catenin destruction complex), thus promoting axin 2 stabilization, and as a consequence β-catenin degradation [53]. We first injected the small molecule XAV939 into the vitreous of E3 chick eyes and collected the eyes after 24 hours to assess if the nuclear β-catenin pattern in the CM and RPE was disrupted. Indeed, XAV939 abolished nuclear β-catenin accumulation in the CM, the RPE and the neuroepithelium of E4 chick eyes (Fig. 11 B, D and F) compared to the non-injected contralateral eye (Fig. 11 A, C and E). Furthermore, injection of XAV939 into the optic cup of E4 chick eyes 30 minutes after retinectomy, successfully induced retina regeneration from the CM (6/10 eyes) (Fig. 11 G and I) and RPE (6/10 eyes) (Fig. 11 G and J–L). PBS-treated control eyes did not regenerate (0/10) (Fig. 11 G and H).

## Discussion

The present study revealed the involvement of β-catenin dependent TCF/Lef1 transcriptional activity in the regulation of the stem/progenitor cell niche located in the CM and of the maintenance of the RPE in developing chick eyes, especially upon injury, as well as establishing for the first time its role in regulating chick retina regeneration from activation of stem/progenitor cells and from RPE transdifferentiation. We observed dynamic changes of active nuclear β-catenin protein in early stages of eye development (E1.5–E7, HH stages 10–32). During early eye development, nuclear β-catenin is widespread throughout the optic vesicle and by E4 (HH stage 22–24), nuclear β-catenin positive cells were present in the CM, NE and RPE. This temporal and spatial pattern of nuclear β-catenin in developing chick eyes is consistent with the reported expression pattern of Wnt signaling components Lef1 and activation of Wnt/β-catenin signaling during chick and mouse eye development detected by Lef1/TCF reporter assays [16], [18], [32], [33], [35], [54], [55]. Our finding supports previous reports that identified low proliferative cells in the NPE of chick eyes, specifically after stage 23 (E4). The low proliferating activity of NPE cells was correlated to the onset of expression of chick ciliary epithelial specific genes [56].

It is important to note that while canonical Wnt signaling plays a role in the specification of the CM including the iris and ciliary body in birds and mice [16], [36], the contribution of Wnt signaling on cell proliferation in the CM and/or neuroepithelium/retina has been controversial. For instance, studies in chick eyes manipulating the Wnt signaling pathway at the optic vesicle stage have shown that this pathway is associated with negative regulation of the cell cycle if the analysis is performed after E7.5 [16] vs. a positive regulation when the analysis is performed earlier at E 3.5, or if analyzed in vitro [33], [57]. Similarly in different animal species, the Wnt pathway has been associated with positively regulating the cell cycle in the CM [58], [59] or inhibiting the cell cycle [36]. It is clear that Wnt signaling plays a variety of roles during early eye development and these roles depend on the particular stage, tissue and animal species.

In the differentiated RPE of E4 eyes, we detected reduced nuclear β-catenin immunoreactivity compared to the anterior CM region, but the few nuclear β-catenin^+^ RPE cells were also found to be Sox2^-^, BrdU^-^ and PH3^-^ indicating the low-proliferative state of those cells. This data is consistent with previous reports that showed that the Wnt/β-catenin signaling is activated in the developing RPE and is required for RPE specification [17], [18].

The dynamic changes of nuclear β-catenin staining in the CM in response to retinectomy and during FGF-induced regeneration further suggests that nuclear β-catenin is important to keep the NPE cells (retinal stem/progenitor cells) in a low proliferative state during development (specifically at E4). Moreover, the loss of nuclear β-catenin protein in response to retinectomy is likely to release those cells from their low proliferative state, but not sufficient to induce continuous proliferation. Adding exogenous FGF2 is able to further interrupt β-catenin nuclear accumulation in those NPE cells and induce proliferation to give rise to a new neuroepithelium.

As to the RPE, nuclear β-catenin was detected in differentiating RPE cells at E4; however it was decreased at E5 and older embryos. Upon retinectomy, the significant up-regulation of nuclear β-catenin in the PE and the RPE suggests that it prevents those cells from entering the cell cycle, however, in the presence of FGF2, RPE cells lose nuclear β-catenin and undergo further dedifferentiation and proliferation. Previous work has shown that a precise regulation of Wnt/β-catenin signaling is critical for normal RPE development in mouse. The inactivation of Wnt/β-catenin signaling in developing RPE results in the downregulation of RPE-specific genes Mitf and Otx2 and induction of neural retinal markers Chx10 and Rx, resulting in the conversion of RPE to neural retina. On the other hand, over-activation of Wnt/β-catenin signaling impairs RPE patterning during mouse eye development [17], [18], [19]. In support of Wnt maintaining the RPE quiescent, we have shown that when newt retina-less eye cups are injured and cultured with Wnt signaling inhibitor Dickkopf1 (Dkk1), there is an increase in cell proliferation at the edges of the injured RPE [60]. Taken together, our results suggest that both the retinal stem/progenitor cells of CM and cells of the RPE respond to injury by changing their nuclear β-catenin patterns and their proliferative status.

The disruption of β-catenin nuclear distribution by XAV939 in developing chick eyes and the induction of retina regeneration by XAV939 as well as by the retroviral DN-Lef1construct in the absence of FGF2 strongly suggest that the canonical Wnt pathway is involved in both modes of retina regeneration. Canonical Wnt signaling regulates stem and progenitor cell fate and proliferation in other systems [48], [58], [61], [62], [63], [64] and it has been implicated during regeneration in amphibians [65], [66] and fish [58], [67], [68], [69], and recently suggested to play an important role during mammalian regeneration [62], [63], [64], [70], [71], [72], [73], [74] supporting our data. Interestingly, three recent papers on planaria regeneration reveal that inhibiting the canonical Wnt pathway rescues regenerative ability in different head-regeneration deficient species of planaria: *D. lacteum*, *P. kawakatsui* and *P. fluviatilis* [75], [76], [77]. The findings of these studies are consistent with our data.

**Figure 12:**
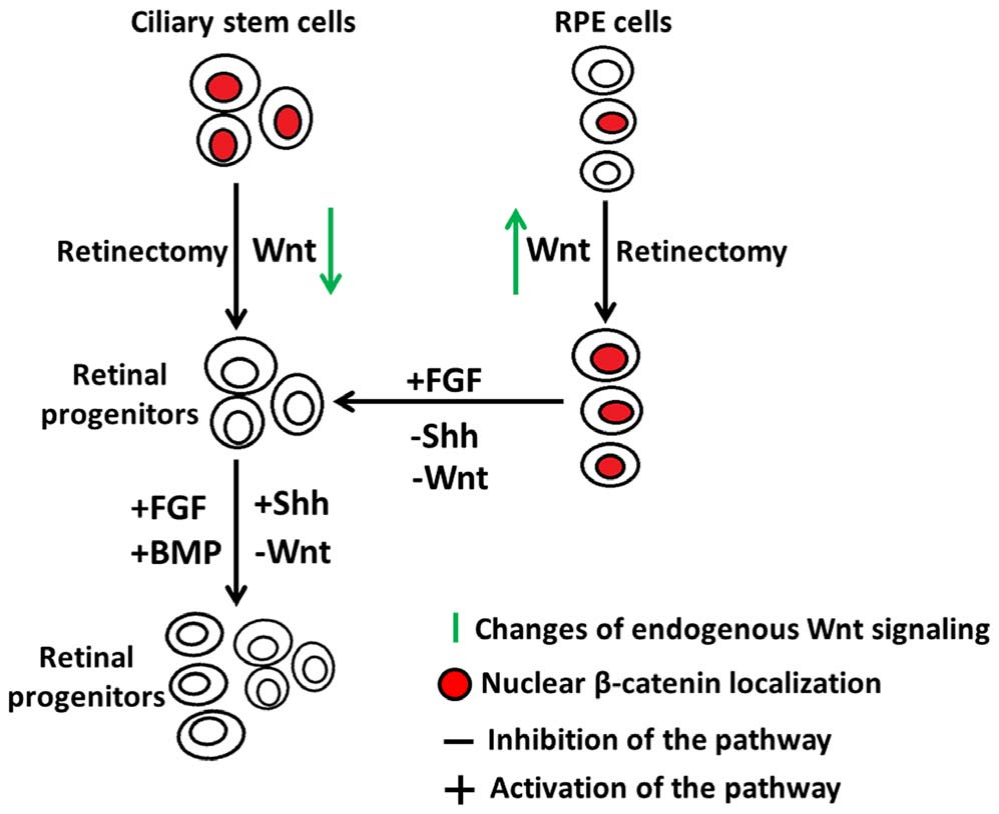
A model depicting the role of active nuclear β-catenin during chick retina regeneration. Nuclear accumulation of β-catenin is transiently lost in the ciliary stem cells of E4 chick eyes after retinectomy to facilitate the cells to respond to inducing factors. Inhibition of Wnt signaling, or overexpression of FGF, Shh or BMP signaling induces ciliary retinal stem/progenitor cells to proliferate and give rise to a new retina. The active nuclear β-catenin in the RPE cells of E4 chick eye is maintained and up-regulated after retinectomy to prevent the RPE cells from entering the cell cycle. Inhibition of Wnt or Shh signaling, or overexpression of FGF signaling induces RPE cells to transdifferentiate into retinal progenitors to further generate a new retina.

In summary, our study provides important information on the regulation of β-catenin activation to maintain retinal stem/progenitor cells and RPE cells, and its pivotal role during the process of injury and retina regeneration (summarized in Fig 12). These results contribute to the better understanding of the regeneration process that could be applied to different organism including mammals.

## Supporting Information

Figure S1 **Nuclear β-catenin^+^ cells in E7 ciliary body (CB) coincide with Collagen IX^+^ area.** (A) Collagen IX labels the CB of the developing chick eye at E7. (B) β-catenin immunostaining of a neighboring section of the same chick eye shows that nuclear β-catenin^+^ cells located in the NPE of the CB, overlap with the Collagen IX^+^ domain. Scale bar in (A) represents 100 µm and applies to (B).
(TIF)

Figure S2 **Pax6 and Chx10 co-localization identifies retinal progenitor cells in DN-Lef1 induced neuroepithelium/retina.** (A–B) The majority of cells in DN-Lef1 induced neuroepithelium from the CM (A) and RPE transdifferentiation (B) at 3 d PR co-express Pax6 and Chx10, indicating their retinal progenitor identity. (C–D) At 7 d PR, there is still a region of retinal progenitors in DN-Lef1 induced neuroepithelium/retina from the CM (C) and from the transdifferentiated RPE (D). (E–H) Pax6/Chx10 double immunostaining on FGF2-induced neuroepithelium/retina from the CM (E, G) and RPE transdifferentiation (F, H) at 3 d and 7 d PR are used for comparison. Cr = ciliary regeneration; Td = transdifferentiation; RPE: retinal pigment epithelium. Scale bar in (A) represents 100 µm and applies to all panels.
(TIF)

File S1 **Supporting tables.** **Table S1**, Primer sequences utilized for RCAS construction and RT-PCR detection. **Table S2**, Primary antibodies utilized for immunohistochemistry experiments. **Table S3**, Primer sequences utilized for RT-qPCR.
(DOCX)
