# Supplementary figures and images for: β-Catenin Inactivation Is a Pre-Requisite for Chick Retina Regeneration
Source: PLoS One. 2014 Jul 8;9(7):e101748. doi: 10.1371/journal.pone.0101748 (PMC4086939; doi:10.1371/journal.pone.0101748)

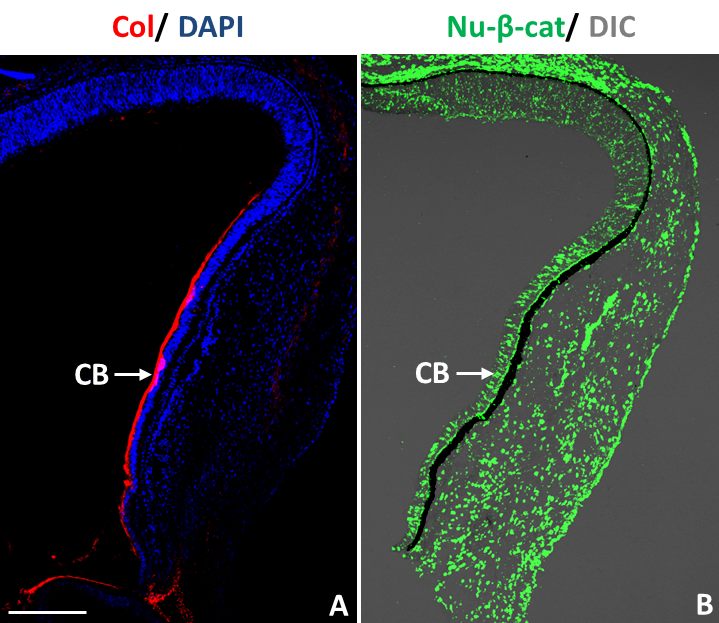

Supplement: Figure S1 — Nuclear β-catenin+ cells in E7 ciliary body (CB) coincide with Collagen IX+ area. (A) Collagen IX labels the CB of the developing chick eye at E7. (B) β-catenin immunostaining of a neighboring section of the same chick eye shows that nuclear β-catenin+ cells located in the NPE of the CB, overlap with the Collagen IX+ domain. Scale bar in (A) represents 100 µm and applies to (B). (TIF) [file pone.0101748.s001.tif]

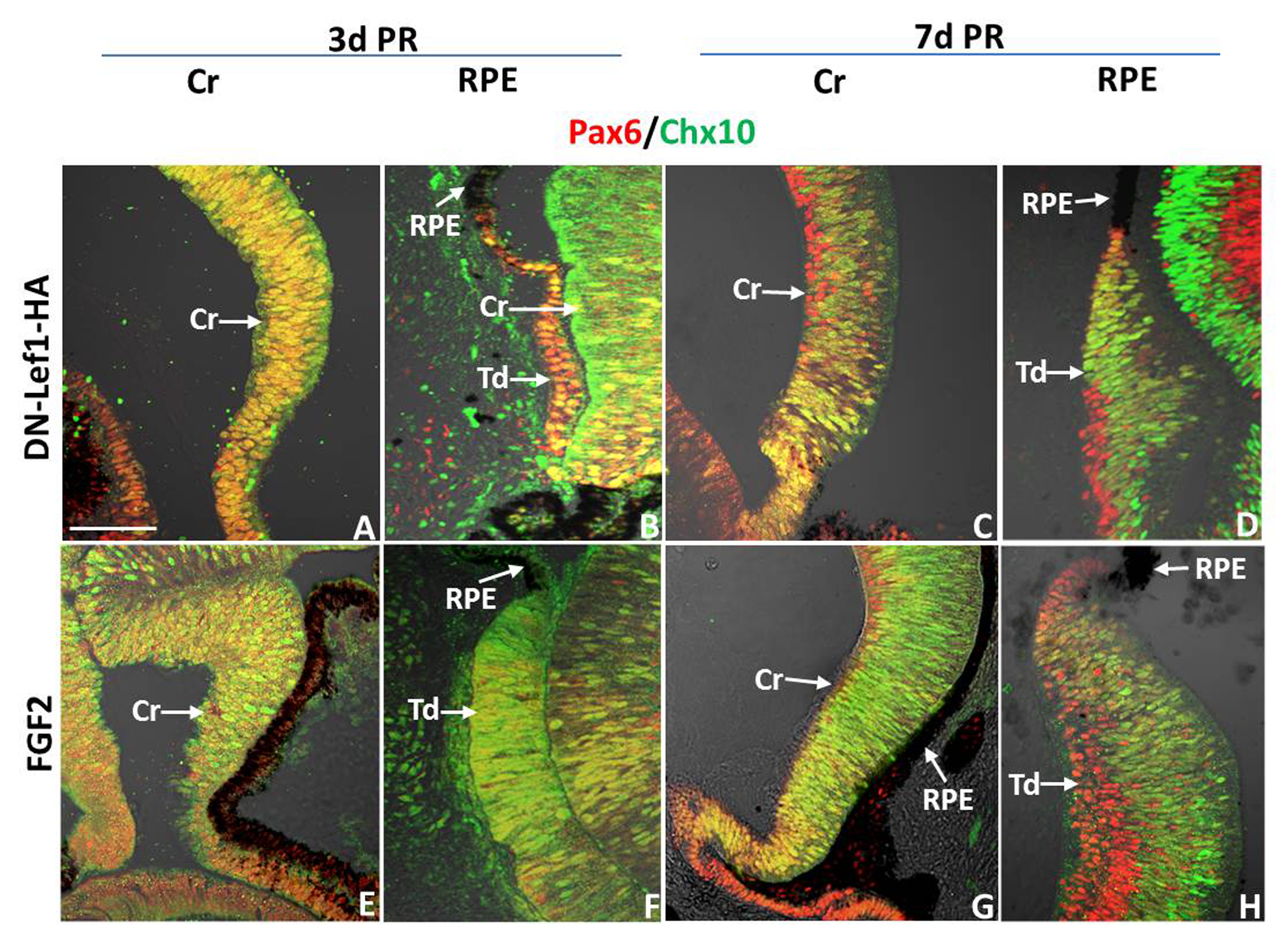

Supplement: Figure S2 — Pax6 and Chx10 co-localization identifies retinal progenitor cells in DN-Lef1 induced neuroepithelium/retina. (A–B) The majority of cells in DN-Lef1 induced neuroepithelium from the CM (A) and RPE transdifferentiation (B) at 3 d PR co-express Pax6 and Chx10, indicating their retinal progenitor identity. (C–D) At 7 d PR, there is still a region of retinal progenitors in DN-Lef1 induced neuroepithelium/retina from the CM (C) and from the transdifferentiated RPE (D). (E–H) Pax6/Chx10 double immunostaining on FGF2-induced neuroepithelium/retina from the CM (E, G) and RPE transdifferentiation (F, H) at 3 d and 7 d PR are used for comparison. Cr = ciliary regeneration; Td = transdifferentiation; RPE: retinal pigment epithelium. Scale bar in (A) represents 100 µm and applies to all panels. (TIF) [file pone.0101748.s002.tif]
