# Supplementary material for: β-Catenin Inactivation Is a Pre-Requisite for Chick Retina Regeneration
Source: PLoS One. 2014 Jul 8;9(7):e101748. doi: 10.1371/journal.pone.0101748 (PMC4086939; doi:10.1371/journal.pone.0101748)
Supplement: File S1 — Supporting tables. Table S1, Primer sequences utilized for RCAS construction and RT-PCR detection. Table S2, Primary antibodies utilized for immunohistochemistry experiments. Table S3, Primer sequences utilized for RT-qPCR. (DOCX) [file pone.0101748.s003.docx]

**Tables**

**Table S1.** **Primer sequences utilized for RCAS construction and RT-PCR detection**

RCAS DN-Lef1-HA construction (5’-3’)

F: CACC**ATG**gagaaggagaagatcttcg

R: AGCTGGGTTgatgtaggcagctgtcat

RT-PCR detection

F: aggcagctacacatgcagc

R: GCATAATCTGGCACGTCATAAGGG

**F: forward primer; R: reverse primer. Lowercase sequences belong to Lef1 sequence (NM_205013) and ATG codon is bold**

**Table S2.** **Primary antibodies utilized for immunohistochemistry experiments**

| **Antibody** | **Dilution** | **Vendor** |
| --- | --- | --- |
| Anti PY-489-β-catenin  Anti-viral gag (AMV3C2)  Anti-Pax6  Anti-Napa 73 (E/C8)  Anti-visinin (7G4)  Anti-vimentin (H5)  Anti-AP2 (3B5) | 1:20  1:100  1:10  1:100  1:100  1:100  1:100 | Developmental Studies Hybridoma Bank  (Iowa City, Iowa, USA) |
| Anti-Prox1 | 1:2000 | Millipore (Billerica, MA, USA) |
| anti-Brn3a | 1:100 |  |
| Anti-Chx10 | 1:50 | Exalpha (Shirley, MA, USA) |
| Anti-BrdU | 1:100 | Abcam (Cambridge, MA, USA) |
| Anti-Sox2 (D17) | 1:50 | Santa Cruz (Dallas, Texas, USA) |
| Anti-p27 (Kip1) | 1:50 | BD Biosciences (San Jose, CA, USA) |

**Table S3. Primer sequences utilized for RT-qPCR**

Gene Ensembl ID (5’-3’) Primers efficiency

(slope / % efficiency)

*cD1* ENSGALT00000012217 F: ACCGACAACTCCATCAGACC -3.56 / 91.0

R: ATTGCAGCCAGATTCCATTT

*Msi1* ENSGALG00000007227 F: CCCTGAGTTCCGTGTAGAGC -3.52 / 92.5

R: GTGAGGGGAATTGCTGTGAG

*Gapdh* ENSGALG00000014442 F: CCATGTTTGTGATGGGTGTC -3.63 / 88.5

R: CTCCACAATGCCAAAGTTGT

**F: forward primer; R: reverse primer**
